# Platelet-Derived Exosome Product Prolongs Stem Cell-Derived β-Cell Graft Survival and Is Associated with Reduced Nk Cell Infiltration and Immunomodulation

**DOI:** 10.3390/cells15151367

**Published:** 2026-07-29

**Authors:** Zenith Khashim, Swikriti Shrestha, Shaimaa Hassoun, Ethan W. Law, Anna Marie R. Schornack, Jessica Lacap, Danielle J. Beetler, Gabriel J. Weigel, Lauren T. Jennings, Laura Becher, Chris Paradise, Atta Behfar, DeLisa Fairweather, Quinn P. Peterson

**Affiliations:** 1 Department of Physiology and Biomedical Engineering, Mayo Clinic, Rochester, MN 55905, USAanna.marie.r.schornack@vanderbilt.edu (A.M.R.S.); lacap2236@gmail.com (J.L.); 2 Mayo Clinic Graduate School of Biomedical Sciences, Mayo Clinic, Rochester, MN 55905, USA; 3 Department of Cardiovascular Medicine, Mayo Clinic, Jacksonville, FL 32224, USA; 4 Rion Inc., Rochester, MN 55902, USA; 5 Department of Cardiovascular Disease, Mayo Clinic, Rochester, MN 55905, USA; 6 Van Cleve Cardiac Regenerative Medicine Program, Mayo Clinic, Rochester, MN 55905, USA; 7 Department of Immunology, Mayo Clinic, Jacksonville, FL 32224, USA

**Keywords:** stem cell-derived β cells, platelet derived exosome product, type 1 diabetes, xenograft rejection, natural killer cells, immunomodulation, graft survival

## Abstract

**Highlights:**

**What are the main findings?**

- PEP delayed SC-β cell xenograft rejection and reduced NK-cell infiltration in immunocompetent mice.
- A PEP reduced NK-cell-associated gene expression in stimulated PBMCs and modulated inflammatory pathways in vivo.

**What are the implications of the main findings?**

- PEP may serve as an adjunct immunomodulatory agent to improve SC-β cell graft survival in type 1 diabetes.
- Targeting NK-cell-associated responses may help reduce immune-mediated loss of stem cell-derived β cells.

**Abstract:**

Stem cell-derived β (SC-β) cells are a promising therapy for type 1 diabetes (T1D), but their long-term efficacy is limited by immune-mediated graft rejection. Platelet-derived exosome product (PEP) has emerged as a novel immunomodulatory agent, although its effect on SC-β cell xenograft rejection remains unclear. Here, we evaluated PEP in stimulated human peripheral blood mononuclear cells (PBMCs) and in an immunocompetent mouse model of SC-β cell transplantation under the kidney capsule. Immune-related gene expression was assessed by quantitative PCR, immune cell infiltration by immunofluorescence, and graft function by circulating human insulin. In vitro, PEP reduced NK-cell-associated gene expression, including NK1.1, EOMES, and KLRK1, and increased IL-10 expression. In vivo, untreated SC-β cell xenografts showed progressive immune infiltration and loss of detectable human insulin by day 14, whereas PEP-treated grafts retained detectable insulin through day 14 across co-transplantation, pretreatment, and systemic administration strategies. PEP co-transplantation delayed, but did not prevent, xenograft rejection, with graft loss observed by day 21. This delay was associated with reduced NK1.1+ cell infiltration and lower expression of selected inflammatory and rejection-associated markers, including NK1.1, Nos2, and Nlrp3. These findings suggest that PEP modulates graft-associated immune responses and may serve as an adjunct immunomodulatory strategy for stem cell-based therapies for type 1 diabetes. Further studies are needed to evaluate sustained graft durability, safety, and translational efficacy.

## 1. Introduction

Type 1 diabetes (T1D) is characterized by autoimmune destruction of insulin-producing pancreatic β cells. At present, exogenous insulin administration remains the primary treatment [1]. However, despite major advances in diabetes management, including insulin pumps and continuous glucose-monitoring systems, many patients continue to experience suboptimal glycemic control, long-term complications, and potentially life-threatening episodes of hypoglycemia [2,3]. In recent years, transplantation of human pluripotent stem cell-derived β (SC-β) cells has emerged as a promising therapeutic strategy for T1D. These cells closely resemble native human pancreatic β cells, and early clinical studies have supported their potential for restoring insulin-producing cell mass in patients with diabetes [4,5,6,7]. However, a major obstacle to the broad clinical application of SC-β cell therapies is immune-mediated graft loss. In the allogeneic setting, transplanted SC-β cells may be vulnerable to both allorejection and recurrence of β-cell autoimmunity, whereas autologous SC-β cells may reduce the risk of allorejection but remain susceptible to recurrent autoimmune attack in patients with T1D [7]. Accordingly, systemic immunosuppression or alternative immune-protective strategies may be required to preserve graft survival and function. However, long-term use of immunosuppressive agents carries substantial risks and adverse effects, including increased infectious risk, limiting their acceptability for many individuals with diabetes [8]. Therefore, the development of alternative strategies to protect transplanted SC-β cells from immune-mediated rejection has become a major priority in the field. Current approaches include encapsulation technologies that physically shield grafted cells from immune attack, as well as genetic engineering strategies designed to reduce graft immunogenicity and improve engraftment outcomes [7,8,9,10].

In addition to adaptive immune mechanisms, innate immune pathways, including NK-cell-associated responses, are increasingly recognized as important contributors to graft injury and rejection. NK cells can recognize transplanted cells through missing-self mechanisms resulting from inadequate engagement of inhibitory MHC signals, as well as through activating receptors such as NKG2D/KLRK1 that respond to stress-induced ligands on grafted cells [11,12,13,14]. In xenogeneic and allogeneic transplantation settings, these mechanisms may promote direct cytotoxicity and inflammatory amplification within the graft microenvironment. Therefore, strategies that reduce NK-cell recruitment or activation may provide an important approach to limit early immune-mediated loss of transplanted SC-β cells.

Exosomes and other extracellular vesicles are increasingly recognized as heterogeneous, cell-source-dependent mediators of intercellular communication with potential applications in immune modulation, regenerative medicine, and therapeutic delivery. However, clinical translation of exosome-based products remains limited by challenges related to source variability, scalable manufacturing, product characterization, and reproducibility [15,16].

Exosomes are released by most cell types and carry bioactive cargo, including proteins, lipids, and multiple RNA species that can influence recipient cell behavior. Among the most extensively studied are mesenchymal stem cell (MSC)-derived exosomes, which have been shown to modulate immune responses through mechanisms such as suppression of T-cell activity and promotion of regulatory T-cell development [17,18,19,20]. In the context of T1D, exosome-based approaches have been reported to reduce inflammatory responses and improve graft outcomes following islet transplantation [18]. Despite these encouraging findings, MSC-derived exosomes have not yet achieved broad clinical translation. Platelet-derived exosome product (PEP) represents an attractive alternative with potentially greater translational feasibility [21,22,23]. Platelet-derived exosomes are naturally abundant in blood products, and PEP is closely linked to platelet- and plasma-based therapies with long-established clinical safety profiles. More recently, a concentrated, well-defined, lyophilized PEP preparation generated from human donor platelets has been developed as a candidate therapeutic product [24,25,26]. Although PEP may offer a more practical and scalable platelet-derived exosome-based platform than MSC-derived exosomes, it remains unknown whether PEP can modulate immune responses relevant to SC-β cell transplantation and thereby support graft protection. Addressing this knowledge gap is important because immune-mediated injury remains a major barrier to the clinical translation of SC-β cell therapies.

Preclinical studies have demonstrated anti-inflammatory and tissue-protective effects of PEP in mouse models of osteoarthritis, myocardial infarction, myocarditis, and wound healing [25,27,28,29]. Building on these findings, PEP has advanced to Phase II and III clinical trials for several indications, including knee osteoarthritis, wound healing after skin grafting, and acute myocardial infarction (NCT04327635, NCT04664738, and NCT06463132). In the present study, we investigated whether PEP could modulate immune responses relevant to SC-β cell transplantation. Specifically, we examined the effects of PEP in vitro using human peripheral blood mononuclear cells (PBMCs) and in vivo using a xenograft model of SC-β cell transplantation in immunocompetent mice. In addition, syngeneic and allogeneic graft models were included to provide comparative immune-response contexts and to distinguish baseline graft-associated responses from immune responses driven by donor–recipient mismatch. Our findings indicate that PEP treatment is associated with altered immune responses to multiple stimuli, including transplanted SC-β cells, and that these effects can be achieved through different delivery strategies. Collectively, these results support the potential of PEP as an immunomodulatory adjunct in stem cell-based therapies for T1D and it may reduce dependence on systemic immunosuppressive drugs.

## 2. Materials and Methods

### 2.1. Directed Differentiation of SC-β Cells

Human pluripotent stem cell maintenance and differentiation into stem cell-derived β cells were performed as previously described [5]. Pluripotent stem cells were adapted to a three-dimensional (3D) suspension culture system using 500 mL spinner flasks (Corning Inc., Corning, NY, USA; Cat. No. 3153) and maintained in mTeSR™ medium (STEMCELL Technologies, Vancouver, BC, Canada; Cat.24243)Suspension cultures were initiated by seeding 150 million cells in 300 mL mTeSR medium supplemented with 10 µM Y-27632 (R&D Systems, Minneapolis, MN, USA; Cat. No.4448) and cultured at 70 rpm magnetic stirrers plate (Chemglass Life science, Vineland, NJ, USA; Cat. No. CLS-4100-09) in a humidified incubator at 37 °C with 5% CO_2_. The medium was replaced every 48 h without Y-27632, and cells were passaged every 72 h by dissociation into single cells using Accutase (Sigma-Aldrich, St. Louis, MO, USA; Cat. No. A6964). After passaging, cells were reseeded in fresh mTeSR medium containing Y-27632 to initiate the next culture cycle. SC-β cells were generated using a six-stage stepwise differentiation protocol progressing from definitive endoderm to pancreatic progenitors and ultimately to mature functional SC-β cells, as previously described [5]. SC-β cell identity was confirmed by flow cytometric analysis of C-peptide and NKX6.1 co-expression, and functionality was assessed by glucose-stimulated insulin secretion assays.

### 2.2. Flow Cytometry

Cells were collected for flow cytometric analysis at various stages of differentiation. For preparation of single-cell suspensions, cells were dissociated using TrypLE Express (Thermo Fisher Scientific, Waltham, MA, USA; Cat. No. 12563029) at 37 °C for 10 min in a water bath, followed by quenching with S3 medium. Cells were counted, fixed in 4% paraformaldehyde (PFA) (Millipore Sigma, Burlington, MA, USA; Cat. No. 15710) and stored at 4 °C until staining. For intracellular staining, fixed cells were permeabilized in blocking solution containing PBS, 0.1% Triton X-100, and 5% donkey serum for 45 min at room temperature. Cells were then incubated with primary antibodies diluted in blocking solution for 1 h at room temperature or overnight at 4 °C. After two washes with PBST (phosphate-buffered saline containing 0.1% Triton X-100), cells were incubated with fluorophore-conjugated secondary antibodies diluted in blocking solution for 1 h at room temperature. Cells were washed three times, resuspended in PBST at a concentration of 1 × 10^6^ cells/mL, and analyzed using Attune NxT Acoustic Focusing Cytometer with Cytkick Autosampler (Thermo Fisher Scientific, Waltham, MA, USA). Data were processed using FlowJo v10 and Attune NxT v4.2.6 software. SC-β cell identity was confirmed by flow cytometric analysis of C-peptide (DSHB Iowa city, IA, USA; Cat. No. GN-ID4, dilution of 1:250) and NKX6.1 (DSHB, Iowa city, IA, USA; Cat. No. F55A12, dilution 1:100) co-expression, and functionality was assessed by glucose-stimulated insulin secretion (GSIS) assays. Secondary antibodies included donkey anti-rat Alexa Fluor 488 (Invitrogen, Carlsbad, CA, USA; Cat. No. A-21208; 1:1000) and donkey anti-mouse Alexa Fluor 647 (Invitrogen, Carlsbad, CA, USA; Cat. No. A-31571; 1:1000).

### 2.3. Hormone Secretion Assays (GSIS)

SC-β cell clusters were washed once with 1× PBS and twice with low-glucose (3.3 mM) Krebs–Ringer buffer (KRB). Clusters were then transferred to a 24-well plate fitted with transwell inserts and preincubated in low-glucose KRB for 1 h at 37 °C. After preincubation, clusters were washed again and sequentially incubated in low-glucose KRB (3.3 mM), high-glucose KRB (16.5 mM), and KRB containing 30 mM KCl for 1 h each at 37 °C. After each incubation step, supernatants were collected and stored at −20 °C until analysis. Following the final incubation, SC-β cell clusters were dissociated using 1 mL TrypLE Express and counted using a Vi-CELL automated cell counter. Insulin concentrations in the collected supernatants were measured using a human insulin ELISA kit (ALPCO, Salem, NH, USA; Cat. No. 80-INSHUU-E10) and normalized to the total cell number.

### 2.4. Immunofluorescence Staining

SC-β cell clusters or kidney grafts were fixed in 4% paraformaldehyde (PFA) for 1 h at room temperature or overnight at 4 °C, followed by three washes with 1× PBS. Cell clusters were embedded in Histogel, whereas kidney grafts were paraffin-embedded and sectioned. Paraffin-embedded tissues were deparaffinized using Histo-Clear, rehydrated through a graded ethanol series, and subjected to antigen retrieval by boiling in 10 mM sodium citrate buffer (pH 6.0) for 1 h. After three washes, sections were blocked in PBS containing 0.1% Triton X-100 and 5% donkey serum for 40 min. Slides were then incubated with primary antibodies overnight at 4 °C. Where applicable, graft identity was evaluated using model-specific markers: insulin for xenogeneic grafts, PDX1 for syngeneic grafts, and vimentin for allogeneic grafts. Immune cell infiltration was assessed using antibodies against CD45, CD11b, F4/80, CD11c, Ly6G, CD3, B220, and NK1.1 (Supplementary Table S1). Following primary antibody incubation, slides were washed three times for 15 min each, incubated with appropriate secondary antibodies for 2 h at room temperature, washed three times again, and mounted using Fluoromount-G containing DAPI-4′,6-diamidino-2-phenylindole (Invitrogen, Carlsbad, CA, USA; Cat. No. 00-4959-52). Fluorescent imaging was performed using a ZEISS Axio Observer inverted microscope with an AxioCam 702 and image were acquired using, ZEN 2.5 Blue edition software (Carl Zeiss Microscopy GmbH, Jena, Germany). All images shown are representative of findings observed in at least three independent biological samples.

### 2.5. Quantification of Immunofluorescence-Positive Cells

Four kidney sections from each mouse were stained, and four distinct randomly selected regions were imaged from each section. Positively stained cells were manually quantified using a Zeiss Axio Observer microscope and ZEN2 Blue software by three independent assessors. The number of positive cells in each section was normalized to the area of the kidney capsule.

### 2.6. Cell Culture

For syngeneic transplantation studies, mouse induced pluripotent stem cells miPSCs (ALSTEM, Richmond, CA, USA; Cat. No. iPS02M), derived from the C57BL/6 mouse strain, were cultured on mitotically inactivated CF-1 mouse embryonic fibroblasts (MEFs; Applied StemCell, Milpita, CA, USA; Cat. No. ASF-1201), which were derived from CF-1 outbred mice, using feeder-based culture conditions. MEFs were maintained in DMEM (Corning Cellgro, Corning, NY, USA; Cat. No, 10-103-CV) supplemented with 10% fetal bovine serum (FBS), L-glutamine, sodium pyruvate, non-essential amino acids, and penicillin–streptomycin, with media changes every 24 h. Before using as feeders, MEFs were inactivated with mitomycin C (Sigma-Aldrich, St. Louis, MO, USA; Cat No. M4287) for 3 h. miPSCs were cultured in KnockOut DMEM (Thermo Fisher Scientific, 21985023) supplemented with 2-mercaptoethanol and leukemia inhibitory factor (LIF; Sigma-Aldrich, St. Louis, MO, USA; Cat. No. L5158-10UG). Colonies were collected and replated every 5–7 days, depending on confluency, until differentiation was initiated. Differentiation into mouse pancreatic progenitor cells followed the first three stages of the human SC-β cell differentiation protocol described above. Pancreatic progenitor identity was confirmed by flow cytometry and immunofluorescence for PDX1 expression. To initiate cluster formation, approximately 3 × 10^4^ cells were plated per spheroid well (Corning, Corning, NY, USA; Cat. No: 3830). On day 7, all clusters were harvested and prepared for transplantation.

### 2.7. SC-β Cells Transplantation

All animal procedures were approved by the Mayo Clinic Institutional Animal Care and Use Committee. Male C57BL/6 mice aged 8–10 weeks and weighing 19–22 g were used as transplant recipients. Animals were housed under sterile conditions with ad libitum access to food and water. SC-β cell transplantation under the kidney capsule was performed as previously described [5]. For xenogeneic transplantation, approximately 5 × 10^6^ human SC-β cells were transplanted into the left kidney capsule of each mouse. For syngeneic transplantation, approximately 5 × 10^6^ mouse pancreatic progenitor cells were used. For allogeneic transplantation, approximately 2.5 × 10^6^ mouse MEFs were transplanted. Sham-operated controls received saline injections under the kidney capsule. Following surgery, mice were housed individually. Blood samples were collected by cheek bleed at designated time points for measurement of human insulin levels by ELISA. Kidneys were harvested at multiple time points (days 2, 5, 14, and 21) for immunofluorescence staining and gene expression analysis.

### 2.8. SC-β Cell PEP Treatment

PEP was administered in three different ways in the SC-β cell transplantation experiments. For the pretreatment group, SC-β cells were incubated in vitro with 10% PEP for 24 h at 37 °C and then washed three times with PBS before transplantation.

For systemic administration, mice received 25% PEP diluted in sterile saline to a final volume of 200 µL by intraperitoneal injection for three consecutive days starting on the day of SC-β cell transplantation. For the co-transplantation group, 15 µL of 100% PEP was mixed with SC-β cells at the time of transplantation. Saline-treated animals served as controls. On day 14, blood was collected and kidneys were harvested for immunofluorescence and gene expression analysis.

### 2.9. ELISA

At designated time points (days 2, 5, 14, and 21), mouse blood was collected by cheek bleed. Plasma was isolated from whole blood and used to quantify human insulin levels using a Human Ultrasensitive Insulin ELISA kit (ALPCO, Salem, NH, USA; Cat. No. 80-INSHUU-E10). The same ELISA kit was used to measure insulin secretion during in vitro GSIS assays.

### 2.10. Real-Time PCR

At harvest, mouse kidneys were collected and stored at −80 °C until RNA isolation. Frozen kidneys were thawed, cross-sectioned into halves, homogenized, and lysed using a TissueLyser (Qiagen, Germantown, MD, USA) with 7 mm stainless steel beads in RTL buffer containing 0.5% DX buffer to reduce foaming. Total RNA was isolated and purified using the RNeasy Fibrous Mini Kit, including DNase and proteinase K treatment steps (Qiagen, Germantown, MD, USA; Cat. No. 74704). RNA was eluted in 30 µL RNase-free water. Eluted RNA from both halves of each kidney was pooled by sample and aliquoted. RNA concentration was measured using a NanoDrop spectrophotometer (Thermo Fisher Scientific, Waltham, MA, USA). Total RNA from mouse kidneys was analyzed by quantitative PCR using Assay-on-Demand primers and probe sets with the ABI 7000 TaqMan system (Applied Biosystems, Foster City, CA, USA) after reverse transcription into complementary DNA (cDNA) using a High-Capacity cDNA Reverse Transcription Kit (Applied Biosystems, Foster City, CA, USA; Cat. No. 4368813). Gene expression data are presented as relative gene expression normalized to GAPDH. Relative expressions were calculated using comparative threshold cycle (Ct) analysis. For PBMC and SC-β cell experiments, total RNA was purified using the RNeasy Mini Kit (QIAGEN, Germantown, MD, USA; Cat. No) according to the manufacturer’s instructions. cDNA was synthesized from 400 ng total RNA using reverse transcription according to the manufacturer’s protocol (Qiagen). Quantitative real-time PCR was performed in 20 µL reaction volumes containing 1 µL cDNA, 200 nM of each primer, and 10 µL SYBR Select Master Mix using (Applied Biosystems, Foster City, CA, USA; Cat No. 4472908) using a7500 Real-Time PCR System (Applied Biosystems, Foster City, CA, USA). Primer sequences are listed in Supplementary Table S2.

### 2.11. PBMCS Isolation, Stimulation, and PEP Treatment

Whole blood was obtained from the Mayo Clinic Blood Bank, and PBMCs were isolated using Ficoll-Paque Plus (Cytiva). The buffy coat layer was collected and washed twice with PBS. Freshly isolated PBMCs were cultured in low-attachment tissue culture plates with or without stimulation for 24 h. The experiment included four groups: (1) unstimulated control; (2) stimulation with LPS (Sigma-Aldrich, St. Louis, MO, USA; Cat No. L4391) or anti-CD3/CD28 antibodies (Invitrogen, Carlsbad, CA, USA; Cat. No. 16-0037-81 and 16-0289-81); (3) co-stimulation with LPS or CD3/CD28 in the presence of 10% PEP; and (4) post-stimulation treatment with 10% PEP for 24 h. After incubation, cells were washed with PBS and processed for mRNA isolation for gene expression analysis (Supplementary Table S3).

### 2.12. Statistical Analysis

Data were graphed and analyzed using GraphPad Prism version 10.0.0 (GraphPad Software, San Diego, CA, USA). Comparisons between two groups were performed using an unpaired Student’s *t*-test. For experiments involving multiple groups, one-way or two-way analysis of variance (ANOVA), followed by Tukey’s or Sadak’s multiple-comparison test, as appropriate, was used. Data are presented as mean ± SEM, and a *p* value < 0.05 was considered statistically significant.

## 3. Results

### 3.1. PEP Modulates Human Immune Cell Activity In Vitro

**Figure 1:**
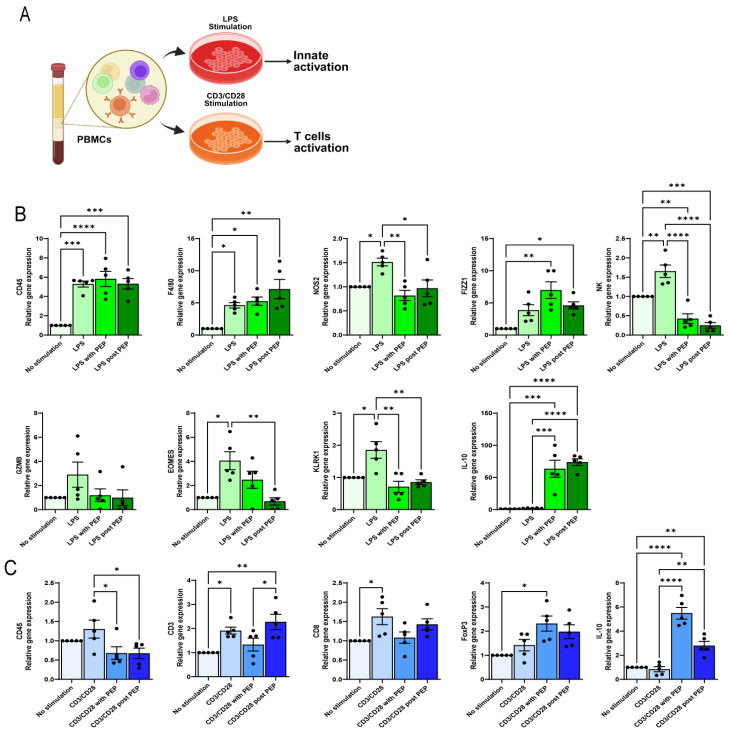
Activation of human PBMCs by LPS or CD3/CD28 stimulation and the immunomodulatory effects of PEP. (**A**) Schematic overview of experimental design. Freshly isolated human PBMCs were stimulated with LPS to activate innate immune responses or with anti-CD3/CD28 antibodies to activate T cells. PEP (10%) was added either concurrently with stimulation or after stimulation. (**B**) Gene expression analysis of PBMCs following LPS stimulation with or without PEP treatment, including CD45, F4/80, NOS2, FIZZ1, NK, GZMB, EOMES, KLRK1, and IL10. (**C**) Gene expression analysis of PBMCs stimulated with anti-CD3/CD28 showing expression of CD45, CD3, CD8, FOXP3, and IL10. All gene expression values were normalized to unstimulated controls and are presented as mean ± SEM. Statistical analysis was performed using two-way ANOVA followed by multiple-comparison test. Data are presented as mean ± SEM. Statistical significance is indicated as * *p* < 0.05, ** *p* < 0.01, *** *p* < 0.001, and **** *p* < 0.0001.

To evaluate the potential of PEP to modulate immune responses relevant to graft rejection, we first examined their effects in a controlled in vitro system using PBMCs. PBMCs were stimulated with either lipopolysaccharide (LPS) to activate innate immune responses, including macrophage- and natural killer (NK)-associated pathways, or anti-CD3/CD28 antibodies to stimulate T cells (Figure 1A). Cells were treated with PEP either concurrently with the stimulus or during the 24 h period following stimulation. At the end of each experiment, cells were collected for quantitative RT-PCR analysis of immune cell-associated marker genes.

We first analyzed the effects of PEP in LPS-stimulated PBMCs. LPS induced a marked increase in the expression of CD45, a pan-leukocyte marker, and this response was not significantly altered by PEP treatment (Figure 1B). Similarly, LPS increased expression of the macrophage-associated marker F4/80 and the pro-inflammatory marker NOS2, whereas the M2-associated marker FIZZ1 was not upregulated by LPS alone. PEP treatment increased FIZZ1 expression and reduced NOS2 expression, suggesting that PEP influences macrophage-associated inflammatory responses under these conditions.

In contrast, markers associated with NK-cell activity were more consistently suppressed by PEP treatment. LPS stimulation increased expression of NK1.1, and this increase was significantly reduced by PEP treatment administered either concurrently with or after LPS stimulation (*p* < 0.01 for both conditions). To further assess this observation, we examined additional NK-cell-associated genes, including GZMB, EOMES, and KLRK1. LPS stimulation increased EOMES and KLRK1 expression and produced a modest increase in GZMB compared with unstimulated controls. Notably, EOMES expression was significantly reduced in the PEP post-treatment group (*p* < 0.01), whereas KLRK1 expression was suppressed in both the co-treatment and post-treatment groups (*p* < 0.01). Together, these findings indicate that PEP treatment is associated with reduced expression of several NK-cell-related genes in stimulated PBMCs. We next assessed the anti-inflammatory cytokine IL-10. Although IL-10 was not induced by LPS stimulation alone, PEP treatment led to a marked increase in IL-10 expression, with induction observed in both the co-treatment and post-treatment conditions. This finding suggests that enhanced anti-inflammatory signaling may contribute to the immunomodulatory effects of PEP.

We then evaluated whether PEP similarly influenced T-cell activation in PBMCs stimulated with anti-CD3/CD28 antibodies. CD3/CD28 stimulation produced a modest increase in CD45 expression, which was reduced by PEP treatment in both co-treatment and post-treatment conditions (Figure 1C). Expression of the T-cell-associated markers CD3 and CD8 was slightly increased following CD3/CD28 stimulation (*p* < 0.05), but these changes were not substantially altered by PEP treatment. FOXP3 expression was not induced by CD3/CD28 stimulation alone; however, a slight increase was observed in the presence of PEP. Similarly, IL-10 expression was not increased by CD3/CD28 stimulation alone but was upregulated following PEP treatment (*p* < 0.001), both in stimulated and unstimulated conditions. Overall, these results suggest that the immunomodulatory effects of PEP in this system are more prominent in NK-cell- and inflammatory-response pathways than in conventional T-cell activation markers.

### 3.2. NK Cells Are Recruited to Sites of Xenogeneic and Allogeneic Graft Rejection in Immunocompetent Mice

**Figure 2:**
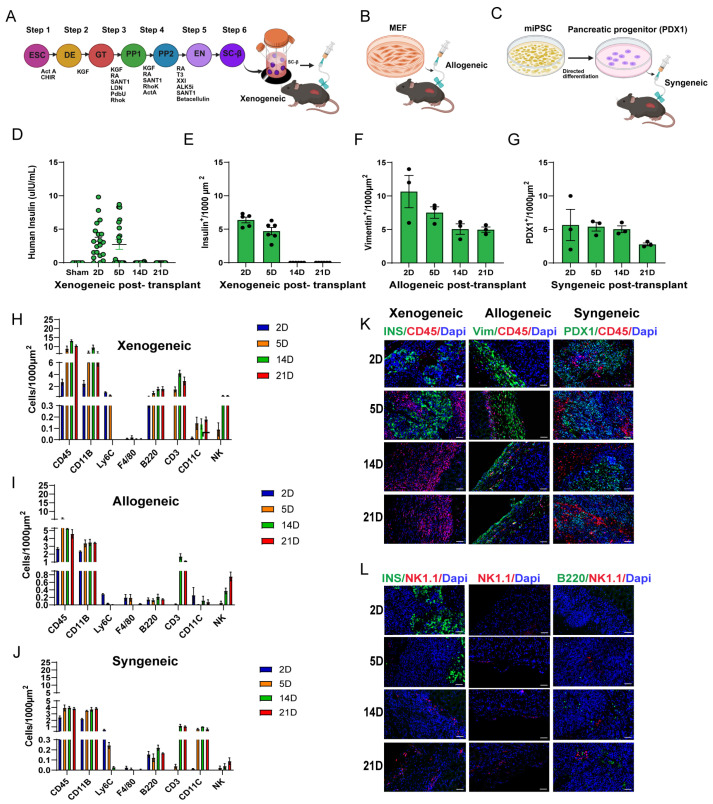
Evaluation of SC-β cells transplantation outcomes and immune cell infiltration in xenogeneic, allogeneic, and syngeneic settings (**A**) Schematic of the six-stage differentiation of human embryonic stem cells into stem cell-derived β cells (SC-β cells), followed by transplantation under the kidney capsule of C57BL/6 recipient mice in a xenogeneic model. (**B**) Allogeneic transplantation model using mouse embryonic fibroblasts (MEFs) as donor cells transplanted into C57BL/6 recipient mice. (**C**) Syngeneic transplantation model using PDX1-expressing pancreatic progenitors derived from C57BL/6 mouse induced pluripotent stem cells and transplanted into C57BL/6 recipient mice. (**D**) Plasma human insulin levels measured in sham-operated mice and xenogeneic recipients at 2, 5, 14, and 21 days after transplantation. (**E**) Quantification of insulin-positive cells in xenogeneic grafts at the indicated time points. (**F**) Quantification of vimentin-positive cells in allogeneic grafts. (**G**) Quantification of PDX1-positive cells in syngeneic grafts. (**H–J**) Quantification of immune cell infiltration in xenogeneic (**H**), allogeneic (**I**), and syngeneic (**J**) grafts, including CD45-positive leukocytes, CD11b-positive myeloid cells, Ly6C-positive cells, F4/80-positive macrophages, B220-positive B cells, CD3-positive T cells, CD11c-positive dendritic cells, and NK1.1-positive natural killer cells. Data are presented as cells per 1000 µm^2^ at 2, 5, 14, and 21 days after transplantation. (**K**) Representative immunofluorescence images of xenogeneic, allogeneic, and syngeneic grafts stained for insulin (green), vimentin (green), or PDX1 (green), respectively, together with CD45 (red) and DAPI (blue). (**L**) Representative immunofluorescence images of xenogeneic grafts stained for insulin (green), NK1.1 (red), and DAPI (blue); allogeneic grafts stained for NK1.1 (red) and DAPI (blue); and syngeneic grafts stained for B220 (green), NK1.1 (red), and DAPI (blue). Images were acquired at 20× magnification, and scale bars represent 50 µm. Figure 2 presents a descriptive analysis of graft persistence and immune cell infiltration over time; no statistical comparisons were performed.

Given the suppression of NK-cell-associated gene expression by PEP in stimulated PBMCs in vitro (Figure 1B), we next examined immune cell recruitment during graft rejection in vivo, with particular attention to NK cells. Although islet allograft rejection is generally considered to be primarily T-cell-mediated, previous studies have suggested that NK cells may also contribute to graft injury and rejection [11,12,13,14]. To investigate this, we established three transplantation models in immunocompetent C57BL/6 mice by implanting cells under the kidney capsule: xenogeneic grafts of human embryonic stem cell-derived SC-β cells (Figure 2A and Figure S1A–E), allogeneic grafts of mouse embryonic fibroblasts (MEFs) (Figure 2B and Figure S1F), and syngeneic grafts of pancreatic progenitor cells derived from mouse induced pluripotent stem cells (miPSCs) (Figure 2C and Figure S1G–I). Mouse pancreatic progenitor cells were used in the syngeneic setting because robust protocols for differentiation of miPSCs into fully mature insulin-producing β-like cells are not yet well established.

Graft-bearing kidneys were collected on days 2, 5, 14, and 21 after transplantation and analyzed by immunofluorescence to assess graft persistence and immune cell infiltration. SC-β cell xenografts underwent rapid rejection, with insulin-positive cells becoming undetectable by day 14 and circulating human insulin levels falling below the detection limit by day 14 after transplantation (Figure 2D,E,K). In contrast, both syngeneic and allogeneic graft remained detectable at day 21, indicating partial or delayed rejection in these models (Figure 2F,G,K).

All three graft types showed evidence of immune cell infiltration, as indicated by CD45 staining (Figure 2H–K). However, the magnitude and kinetics of infiltration differed across models. In syngeneic grafts, CD45+ cell infiltration remained relatively low and stable throughout the 21-day observation period (Figure 2J,K). In contrast, both xenogeneic and allogeneic grafts exhibited more substantial immune infiltration, with CD45+ cell numbers increasing between day 2 and days 5–14 and then declining modestly by day 21 (Figure 2H,I,K). Among these models, infiltration was most pronounced in xenografts, which showed approximately two- to threefold higher CD45+ cell densities per unit graft area than the allogeneic and syngeneic grafts.

To further define the infiltrating immune populations, we performed immunophenotypic analysis of graft sections. Neutrophils (Ly6G+) were recruited early after transplantation in all three models and declined by day 14. Macrophages (F4/80+) were present at relatively low levels across graft types and time points. In contrast, NK1.1+ cells were preferentially enriched in xenogeneic and allogeneic grafts, appearing later than neutrophils and reaching their highest levels at days 14–21 (Figure 2L). B220+ B cells were uncommon in the syngeneic and allogeneic models but accumulated in xenografts at later time points. CD3+ T cells were detected in all three graft settings, although their infiltration appeared earlier and was more pronounced in xenografts than in allogeneic or syngeneic grafts (Figure 2H–K and Figure S1E,F,I). Together, these findings demonstrate dynamic immune cell recruitment to graft sites, with particularly prominent NK-cell accumulation in xenogeneic and allogeneic grafts. These observations support the interpretation that NK cells may contribute to graft rejection in these models and provided the rationale for subsequent experiments testing whether PEP-mediated immune modulation could improve graft survival.

### 3.3. Co-Transplantation of PEP with SC-β Cells Delays NK Cell Recruitment and Xenograft Rejection

Based on our in vitro findings that PEP reduced NK-cell-associated gene expression and increased IL-10 expression in stimulated PBMCs, together with our observation that SC-β xenograft rejection was accompanied by recruitment of multiple immune cell populations, including NK cells, we next asked whether co-transplantation of PEP with SC-β cells could alter immune cell infiltration and prolong graft survival. To address this, SC-β cells were mixed with PEP prior to transplantation under the kidney capsule of immunocompetent mice. Blood samples were collected on days 2, 5, 14, and 21 after transplantation to measure circulating human insulin, and graft-bearing kidneys were harvested for assessment of insulin-positive cell persistence and immune cell infiltration.

**Figure 3:**
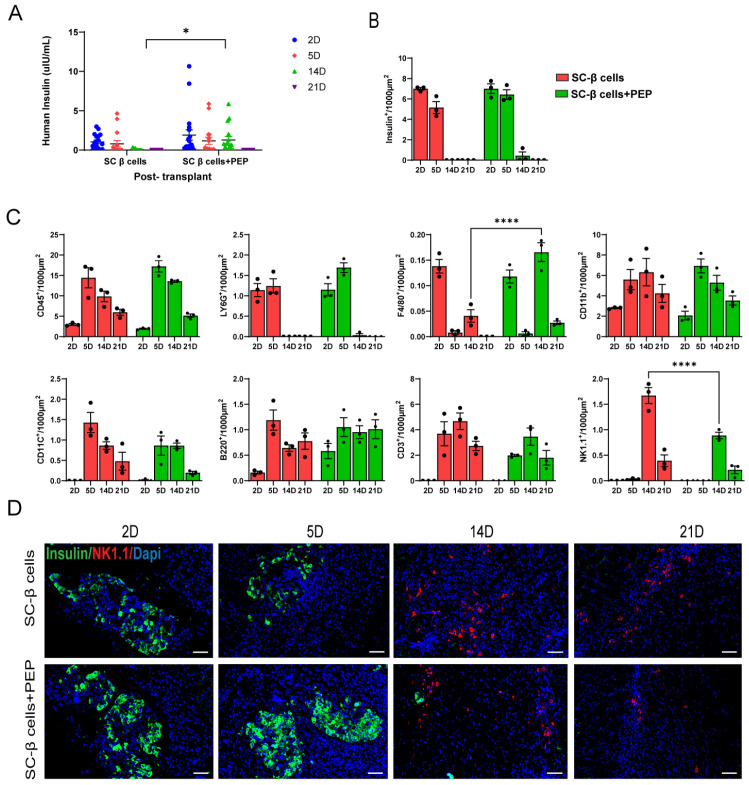
Co-transplantation of PEP with SC-β cells delays NK-cell recruitment and xenograft rejection. (**A**) Human insulin levels in plasma from C57BL/6 mice measured by human-specific insulin ELISA at days 2, 5, 14, and 21 post-transplantations. (**B**) Quantification of insulin + cells within the left kidney capsule at various time points following SC-β cell transplantation. (**C**) Immune cell infiltration in xenogeneic grafts was quantified based on marker expression for CD45^+^ leukocytes, Ly6G^+^ neutrophils, F4/80^+^ macrophages, CD11b^+^ myeloid cells, CD11c^+^ dendritic cells, B220^+^ B cells, CD3^+^ T cells, and NK1.1^+^ natural killer cells. (**D**) Representative immunofluorescence images showing insulin (green) and NK1.1 (red) staining, with nuclei counterstained with DAPI (blue). Images were acquired at 20× magnification; scale bars represent 50 μm. Data are presented as mean ± SEM. Statistical analysis was performed using unpaired Student’s *t*-test for two-group comparisons and two-way ANOVA followed by multiple-comparison testing for multi-group comparisons. Statistical significance is indicated as * *p* < 0.05, **** *p* < 0.0001.

We first compared graft survival in mice transplanted with SC-β cells alone or with SC-β cells plus PEP. Circulating human insulin levels were similar between the two groups on days 2 and 5 after transplantation. By day 14, however, human insulin was no longer detectable in mice transplanted with SC-β cells alone, whereas it remained detectable in mice that received SC-β cells together with PEP (*p* < 0.05; Figure 3A). By day 21, human insulin was no longer detectable in either group, indicating eventual graft loss in both conditions. Immunofluorescence analysis of graft-bearing kidneys was consistent with these findings: insulin-positive cells were detected in both groups at days 2 and 5, but by day 14 only a small number of insulin-positive cells remained, and these were observed exclusively in kidneys from mice co-transplanted with PEP (Figure 3B,D). No insulin-positive cells were detected on day 21 in either group. Together, these findings indicate that co-transplantation of PEP delayed but did not prevent xenograft rejection in this model, suggesting that a single incorporation at the time of transplantation provided only transient graft protection.

We next examined immune cell infiltration in grafts from both treatment groups by immunofluorescence. Overall leukocyte recruitment, assessed by CD45 staining, showed similar temporal patterns in the two groups and was not significantly altered by PEP co-transplantation (Figure 3C,D and Figure S2A,B). We therefore assessed individual immune cell populations to determine whether PEP treatment was associated with selective changes in immune infiltration. Most infiltrating immune cell populations, including Ly6G+ neutrophils, CD11c+ dendritic cells, CD11b+ myeloid cells, B220+ B cells, and CD3+ T cells, showed broadly similar patterns in both groups. In contrast, F4/80+ macrophages were increased at day 14 in the PEP-treated group relative to the SC-β-only group (*p* < 0.0001), indicating a selective difference in macrophage accumulation at this time point. The most notable difference was observed for NK1.1+ cells. At day 5 after transplantation, very few NK cells were detected in SC-β-only grafts, and none were detected in grafts co-transplanted with PEP. By day 14, NK-cell infiltration increased markedly in SC-β-only grafts. In contrast, NK-cell infiltration remained significantly lower in the PEP co-transplant group (*p* < 0.0001; Figure 3C,D). These findings show that co-transplantation of PEP was associated with reduced NK-cell infiltration during the period in which graft persistence was prolonged.

To compare the effects of PEP with a conventional immunosuppressive strategy, we also evaluated SC-β xenografts in mice treated with rapamycin. In rapamycin-treated animals, circulating human insulin remained detectable and insulin-positive cells were present at day 14, whereas untreated grafts had been rejected by this time point (Figure S2C–F). On day 5, NK cells were sparse in both rapamycin-treated and control grafts. By day 14, NK-cell infiltration had increased in both groups but remained lower in rapamycin-treated grafts. By day 21, NK-cell numbers were higher in the rapamycin-treated group than in untreated controls (Figure S2D). In contrast, NK-cell infiltration remained low in the PEP-treated grafts at this time point. Together, these results indicate that both PEP and rapamycin delayed early xenograft rejection and were associated with reduced NK-cell infiltration at intermediate time points. In the PEP co-transplant group, reduced NK-cell infiltration persisted later into the observation period. However, the lower NK-cell infiltration observed on day 21 in the PEP co-transplant group suggests that the temporal effects of PEP on the graft-associated immune response may differ from those of short-term rapamycin treatment.

### 3.4. PEP Delays Xenograft Rejection and Limits Nk Cell Recruitment Across Multiple Administration Routes

**Figure 4:**
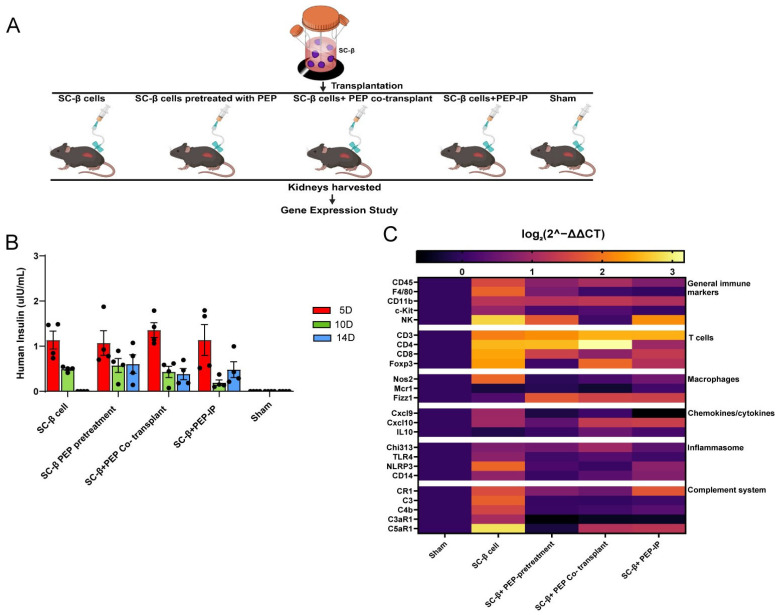
PEP delays xenograft rejection and limits NK-cell recruitment across multiple administration routes. (**A**) Schematic illustration of SC-β cell transplantation with different PEP administration strategies. Group 1: SC-β cells only; Group 2: SC-β cells pretreated in vitro with 10% PEP for 24 h before transplantation; Group 3: SC-β cells co-transplanted with 100% PEP (15 µL); Group 4: systemic PEP delivery via intraperitoneal (IP) injection for 3 days (25% PEP, 200 µL); Group 5: saline control. (**B**) Human insulin levels in plasma from C57BL/6 mice at the indicated time points following transplantation, measured by human-specific insulin ELISA. Data are presented as mean ± SEM. Statistical comparisons were performed among treatment groups at each time point; no statistically significant differences were detected. (**C**) Heatmap of relative mRNA expression of selected immune-related genes in graft-bearing kidneys harvested on day 14. Genes are organized by functional category, including general immune markers, T-cell-associated markers, macrophage-associated markers, chemokines/cytokines, inflammasome-related genes, and complement-associated genes. Expression values are shown as log_2_ (2^−ΔΔCT^) relative to sham controls.

The mechanisms by which PEP modulates immune responses are not yet fully understood. Previous studies using this product have shown beneficial effects following both local and systemic administration [30,31,32,33]. To determine whether PEP could similarly delay xenograft rejection across different dosing strategies, we compared three approaches: co-transplantation of PEP with SC-β cells, pretreatment of SC-β clusters with PEP prior to transplantation, and systemic intraperitoneal administration of PEP on the day of SC-β cell transplantation (Figure 4A). Mice receiving sham surgery served as negative controls. Graft function was assessed by measuring human insulin levels in recipient plasma on days 5, 10, and 14 after transplantation. On day 14, animals were sacrificed and graft-bearing kidneys were collected for gene expression analysis. This approach allowed us to evaluate a broader panel of immune-related markers and to independently assess the observations from the preceding experiments. Consistent with earlier findings, mice transplanted with SC-β cells alone showed detectable human insulin at days 5 and 10 after transplantation, with levels falling below the detection limit by day 14. In contrast, human insulin remained detectable on day 14 in all three PEP-treated groups, regardless of the route of administration. No significant differences in human insulin levels were observed among the three PEP treatment groups (Figure 4B). As expected, no human insulin was detected in sham treated mice at any time point, confirming the specificity of the human insulin ELISA. Together, these findings indicate that PEP treatment delayed xenograft rejection in this model across all three administration routes.

We next analyzed mRNA isolated from graft-bearing kidneys to assess expression of immune-related markers, including pan-immune markers (CD45, F4/80, CD11b, c-Kit), NK-cell markers (NK1.1), T-cell markers (CD3, CD4, CD8, Foxp3), macrophage-associated genes (Nos2, Mrc1, Fizz1), chemokines and cytokines (Cxcl10, Cxcl9, IL-10), inflammasome-related genes (Chi3L3,Tlr4, Nlrp3, CD14), and complement-related genes (CR1,C3,C4b, C3aR1,C5aR1) (Figure 4C and Figure S3A). Saline-treated sham mice were used as the reference group for comparison. Among the pan-immune markers examined, CD45 was significantly elevated in transplanted mice relative to sham controls, whereas the other markers showed no clear differences.

Because our earlier results pointed to NK cells as a prominent immune population associated with graft rejection and PEP responsiveness, we next examined expression of NK1.1. Consistent with previous findings, NK1.1 expression was significantly increased in mice receiving SC-β cell transplants relative to sham controls. This increase was reduced in the PEP-treated groups, with the strongest reduction observed in the co-transplantation group (*p* < 0.05). In contrast, although PEP markedly increased IL-10 expression in vitro, IL-10 expression in vivo was largely unchanged, with only a modest increase observed in the co-transplantation group (*p* < 0.05). These findings suggest that the in vivo effects of PEP on graft survival are not accompanied by broad induction of IL-10 expression at the time point examined.

Compared with sham-operated controls, mice receiving SC-β cell transplants showed increased expression of the pan-T-cell marker CD3, consistent with greater T-cell presence at the graft site. CD4 expression was also elevated across transplant groups. CD8 and Foxp3 showed more variable patterns. Both markers were increased in SC-β-transplanted mice relative to sham controls (*p* < 0.01). In PEP-treated groups, Foxp3 expression was not significantly elevated and was reduced in the PEP pretreatment group, whereas CD8 expression was reduced in the co-transplantation group. These findings indicate that PEP treatment was associated with changes in T-cell-related gene expression, although the direction and magnitude of these effects varied by treatment strategy.

To assess macrophage-associated inflammatory responses in the graft microenvironment, we examined expression of the M1-associated gene Nos2 and the M2-associated genes Mrc1 and Fizz1. Nos2 expression was significantly increased in the SC-β transplant group relative to sham controls, consistent with a pro-inflammatory response, and this increase was reduced in PEP-treated grafts. In contrast, Mrc1 and Fizz1 expression remained relatively unchanged across groups. These findings indicate that PEP treatment was associated with reduced Nos2 expression in vivo, without a corresponding increase in the M2-associated markers examined.

We also assessed inflammatory signaling by measuring selected chemokines and cytokines in graft-bearing kidneys. Cxcl10 and Cxcl9, which are associated with inflammatory cell recruitment, were not significantly altered across groups. As noted above, IL-10 expression showed only a modest increase in the co-transplantation group. Overall, these results suggest that PEP treatment did not broadly alter expression of the chemokines analyzed at this time point. Finally, we examined markers related to inflammasome and complement activation, both of which are implicated in graft rejection [34,35,36]. Expression of the inflammasome-associated gene Nlrp3 was increased in mice transplanted with SC-β cells and was reduced by PEP treatment across all three administration routes (*p* < 0.05). The complement-associated genes CR1, C3, C4b, C3aR1, and C5aR1 showed a similar overall pattern, with higher expression in untreated xenografts and lower expression in PEP-treated groups. Together, these findings indicate that PEP treatment was associated with modulation of multiple immune-related pathways in SC-β xenografts across several routes of administration.

## 4. Discussion

In this study, we show that PEP modulates immune responses in both in vitro and in vivo models of SC-β cell transplantation. Across these experiments, NK-cell-associated responses emerged as a prominent feature of xenograft rejection and as a consistent component of the immune response affected by PEP treatment. In vitro, PEP reduced the expression of several NK-cell-associated genes in stimulated PBMCs, while in vivo PEP treatment was associated with delayed graft rejection and reduced NK-cell infiltration. Together, these findings support the potential of PEP as an immunomodulatory adjunct for improving SC-β cell graft survival. Our in vitro experiments using human PBMCs showed that PEP altered immune-associated gene expression in a selective manner. Although PEP treatment had limited effects on several broad T-cell- and macrophage-associated markers, it reduced LPS-induced expression of NK-cell-associated genes, including NK1.1, EOMES, and KLRK1. Because EOMES is involved in NK-cell maturation and function, and KLRK1 encodes the activating receptor NKG2D, reduced expression of these markers is consistent with altered NK-cell-associated responses following PEP treatment [37,38]. PEP treatment also increased IL-10 expression in vitro, suggesting that PEP may promote an anti-inflammatory signaling environment under these conditions [39]. Notably, some of these effects appeared stronger with co-treatment than with post-treatment, indicating that the timing of PEP exposure may influence the extent of immune modulation.

Using immunocompetent mouse models, we next characterized immune cell recruitment during graft rejection following xenogeneic, allogeneic, and syngeneic transplantation. These studies showed that although our data does not Although our data does not multiple immune cell populations infiltrated graft sites, with particularly prominent recruitment of NK cells in xenogeneic and allogeneic settings at later time points. Because NK cells can recognize cells with altered or mismatched major histocompatibility complex (MHC) signals, their accumulation in these graft models is consistent with a potential contribution to rejection of transplanted cells [40,41,42,43]. Although our data do not establish a direct causal role for NK cells in graft failure, they support the interpretation that NK cells are an important component of the rejection-associated immune response in these models.

To test the therapeutic potential of PEP, we co-transplanted PEP with SC-β cells into immunocompetent mice. Under these conditions, PEP treatment delayed the loss of circulating human insulin and prolonged the persistence of insulin-positive graft cells through day 14, indicating delayed rejection. This effect was accompanied by reduced NK-cell infiltration in PEP-treated grafts relative to untreated controls, consistent with the in vitro PBMC findings. Compared with short-term rapamycin treatment, PEP was also associated with lower NK-cell infiltration at later time points. These observations suggest that PEP and rapamycin may influence graft-associated immune responses differently [44]. The reduction in NK-cell-associated responses following PEP treatment raises the question of whether PEP acts directly on NK cells or indirectly through broader remodeling of the graft immune microenvironment. The present study does not distinguish between these possibilities. PEP may directly alter NK-cell activation, recruitment, or survival. Alternatively, it may indirectly affect NK-cell responses by modulating macrophage-associated inflammatory signaling, cytokine or chemokine networks, or inflammasome/complement-associated pathways within the graft microenvironment. This interpretation is supported by the observed reduction in Nos2 and Nlrp3 expressions in PEP-treated grafts, together with changes in selected immune-related markers. Given the complex cargo of extracellular vesicles, including proteins, RNAs, lipids, and potentially mitochondria or mitochondrial components [45], future studies will be needed to identify the specific PEP-associated cargo responsible for these immunomodulatory effects and to determine whether NK-cell suppression reflects direct vesicle–NK-cell interactions or secondary remodeling of the graft inflammatory environment. This limited durability may, in part, reflect the fact that PEP was incorporated only once at the time of transplantation. We further found that PEP remained associated with delayed xenograft rejection across multiple administration strategies, including co-transplantation, pretreatment of SC-β cells, and systemic intraperitoneal delivery. Across these conditions, graft function at day 14 was preserved relative to untreated controls, and gene expression analysis supported modulation of several immune-related pathways. In addition to reduced NK1.1 expression, PEP-treated grafts showed lower expression of Nlrp3 and Nos2, both of which are associated with inflammatory activation [46,47]. Changes were also observed in selected T-cell-associated markers, including CD8, although these effects varied by treatment strategy. Together, these findings suggest that PEP treatment is associated with broader changes in the inflammatory graft environment in addition to reduced NK-cell-associated responses.

Interestingly, PEP-induced immune modulation differed between the in vitro and in vivo settings. Although PEP strongly increased IL-10 expression in vitro, this pattern was not broadly reproduced in vivo at the time point examined. Conversely, PEP treatment in vivo was associated with reduced expression of the pro-inflammatory marker Nos2, whereas the in vitro PBMC experiments did not show the same macrophage-associated pattern. These differences likely reflect the distinction between the simplified PBMC culture system and the more complex graft microenvironment, where multiple cell types, tissue-derived signals, and treatment timing may shape the overall response to PEP. Although increased F4/80 staining was observed in the SC-β cells + PEP group on day 14, F4/80 is a general macrophage marker and does not distinguish macrophage polarization states. Moreover, PEP did not clearly increase the expression of M2-associated markers, including Fizz1 and Mrc1, suggesting that its effects may be more closely related to dampening inflammatory signaling than to inducing a distinct alternative macrophage program [48,49,50]. Future studies incorporating M2 marker immunostaining will be important to spatially define macrophage phenotypes within the graft microenvironment. Several limitations should be considered. First, the in vivo observation period was limited to 21 days after transplantation; therefore, sustained graft function, long-term immune tolerance, and durability of PEP-mediated protection were not assessed. Second, this study was performed in mouse transplantation models, and species-specific differences in immune responses may affect the translational relevance of these findings to human SC-β cell transplantation. Third, although multiple PEP delivery strategies were evaluated, the optimal dose range, treatment frequency, duration of administration, and safety profile of repeated PEP exposure were not determined. Finally, the active components of PEP and the specific molecular targets responsible for its immunomodulatory effects remain unclear. Future studies should address these limitations through longer follow-up periods, human-relevant immune models, dose–response and safety analyses, and molecular characterization of PEP cargo and target pathways.

Overall, our findings add to growing evidence that innate immune pathways, including NK-cell-associated responses, are important in transplantation settings alongside conventional T-cell-mediated mechanisms [51,52]. Our results show that NK cells are recruited to the graft site and may contribute to graft damage. This expands the traditional understanding of immune responses in transplantation and highlights the importance of targeting both innate and adaptive components of the immune system [53,54,55].

In the context of SC-β cell transplantation, this is particularly relevant because strategies designed to protect grafts from adaptive immune attack may still leave them vulnerable to innate immune recognition. The ability of PEP to delay xenograft rejection across multiple delivery routes therefore supports its potential as an adjunct immunomodulatory agent in stem cell-based therapies for T1D. Future studies will be needed to define the molecular mechanisms by which PEP alters NK-cell-associated responses, determine whether these effects are direct or indirect, and evaluate long-term efficacy in clinically relevant transplantation models.

## 5. Conclusions

This study shows that PEP exerts immunomodulatory effects in the context of SC-β cell transplantation and is associated with delayed xenograft rejection across multiple delivery routes. PEP treatment was consistently linked to reduced NK-cell-associated responses, together with changes in inflammatory pathways within the graft environment. These findings support further investigation of PEP as an adjunct immunomodulatory agent to improve graft survival in stem cell-based therapies for T1D and potentially other transplantation settings.

## Data Availability

The original contributions presented in this study are included in this article/Supplementary Materials. Further inquiries can be directed to the corresponding author.

## Supplementary Materials

The following supporting information can be downloaded at: https://www.mdpi.com/article/10.3390/cells15151367/s1, Figure S1. Characterization of SC-β cells and immune cell infiltration in xenogeneic, allogeneic, and syngeneic settings. (A) Brightfield image showing the clustered morphology of SC-β cells. (B) Flow cytometry plot confirming co-expression of C-peptide and NKX6.1 in differentiated SC-β cells. (C) Glucose-stimulated insulin secretion (GSIS) assay showing increased insulin release under high-glucose (16.5 mM) and KCl (30 mM) conditions compared with low-glucose conditions (3.3 mM). Data are presented as mean ± SEM. (D) Immunofluorescence image of SC-β clusters stained for insulin (green), with nuclei counterstained with DAPI (blue). (E,F,I) Immunofluorescence staining of grafts retrieved from xenogeneic, allogeneic, and syngeneic transplantation models at 2, 5, 14, and 21 days after transplantation. In xenogeneic grafts (E), Ly6G, CD11b, and B220 were shown in red, whereas CD11c, CD3, and F4/80 were shown in white. In allogeneic grafts (F), vimentin was shown in green; Ly6G, F4/80, CD11c, and B220 in red; CD11b in yellow; and nuclei in blue with DAPI. In syngeneic grafts (I), Ly6G, CD11c, and CD11b were shown in green; PDX1 in red; CD3 and F4/80 in white; and nuclei in blue with DAPI. (G) For syngeneic transplantation, mouse iPSCs (miPSCs) were cultured on MEFs, formed spheroids, and stained for OCT4 (green) to confirm pluripotency. Following directed differentiation, pancreatic progenitors were confirmed by PDX1 staining (red). (H) Flow cytometry analysis of differentiated pancreatic progenitors showing the secondary-antibody control, OCT4 expression in pluripotent cells, SOX17 and HNF1β expression at the definitive endoderm stage, and PDX1 expression in pancreatic progenitor cells. Images were acquired at 20× magnification; scale bars represent 50 µm. Figure S2. Co-transplantation of PEP with SC-β cells and SC-β cells transplantations with and without rapamycin injection. (A,B) Representative immunofluorescence images of xenogeneic grafts following co-transplantation of PEP with SC-β cells. Insulin is shown in green; CD45, Ly6G, CD11b, and B220 are shown in red; and CD3, CD11c, and F4/80 are shown in white. (C) Human insulin levels in the plasma of C57BL/6 mice, measured using a human-specific insulin ELISA on days 5, 10, 14, and 21 after transplantation, with or without rapamycin treatment. (D) Quantification of insulin-positive cells and immune-cell markers within the left kidney capsule at the indicated time points following SC-β cell transplantation with or without rapamycin treatment. (E,F) Representative immunofluorescence images of xenogeneic grafts following SC-β cell transplantation with or without rapamycin treatment. Insulin is shown in green; CD45, Ly6G, CD11b, NK1.1, and B220 are shown in red; and CD3, CD11c, and F4/80 are shown in white. Images were acquired at 20× magnification; scale bars represent 50 µm. Statistical analysis was performed using an unpaired Student’s *t*-test for two-group comparisons and two-way ANOVA followed by multiple-comparison testing for multigroup comparisons. Statistical significance is indicated as * *p* < 0.05, ** *p* < 0.01, and *** *p* < 0.001. Figure S3. PEP delays xenograft rejection and limits NK cell recruitment across multiple administration routes. (A) Relative gene expression analysis of immune-related markers in graft-bearing kidneys from sham, SC-β cell, SC-β + PEP pretreatment, SC-β + PEP co-transplant, and SC-β + PEP intraperitoneal (IP) groups. Markers are grouped into general immune markers, T cell–associated markers, macrophage-associated markers, chemokines/cytokines, inflammasome-related genes, and complement system-related genes. Data are presented as relative gene expressions normalized to sham controls and expressed as mean ± SEM. Statistical analysis was performed using one-way ANOVA followed by multiple-comparison testing. Statistical significance is indicated as * *p* < 0.05, ** *p* < 0.01, and *** *p* < 0.001. Table S1. Antibodies used in IF staining. Table S2. RT-PCR primers. Table S3. RT-PCR primers for PBMCs.

## Abbreviations

T1D	Type 1 diabetes
SC-β cells	Stem cell-derived β cells
PEP	Platelet-derived exosome product
PBMCs	Peripheral blood mononuclear cells
NK	Natural killer
IF	immunofluorescence
GSIS	glucose-stimulated insulin secretion
IL-10	Interleukin-10
Nos2	Nitric oxide synthase 2
FIZZ1	Found in inflammatory zone 1
KLRK1	Killer cell lectin-like receptor K1
EOMES	Eomesodermin
GZMB	Granzyme B
MEF	Mouse embryonic fibroblast
miPSC	Mouse induced pluripotent stem cell
hESC	Human embryonic stem cell
MEFs	Mouse embryonic fibroblasts
