# Supplementary material for: Platelet-Derived Exosome Product Prolongs Stem Cell-Derived β-Cell Graft Survival and Is Associated with Reduced Nk Cell Infiltration and Immunomodulation
Source: Cells. 2026 Jul 29;15(15):1367. doi: 10.3390/cells15151367 (PMC13465560; doi:10.3390/cells15151367)
Supplement: Supplementary file 1 [file cells-15-01367-s001.zip › cells-4383135-supplementary.pdf]

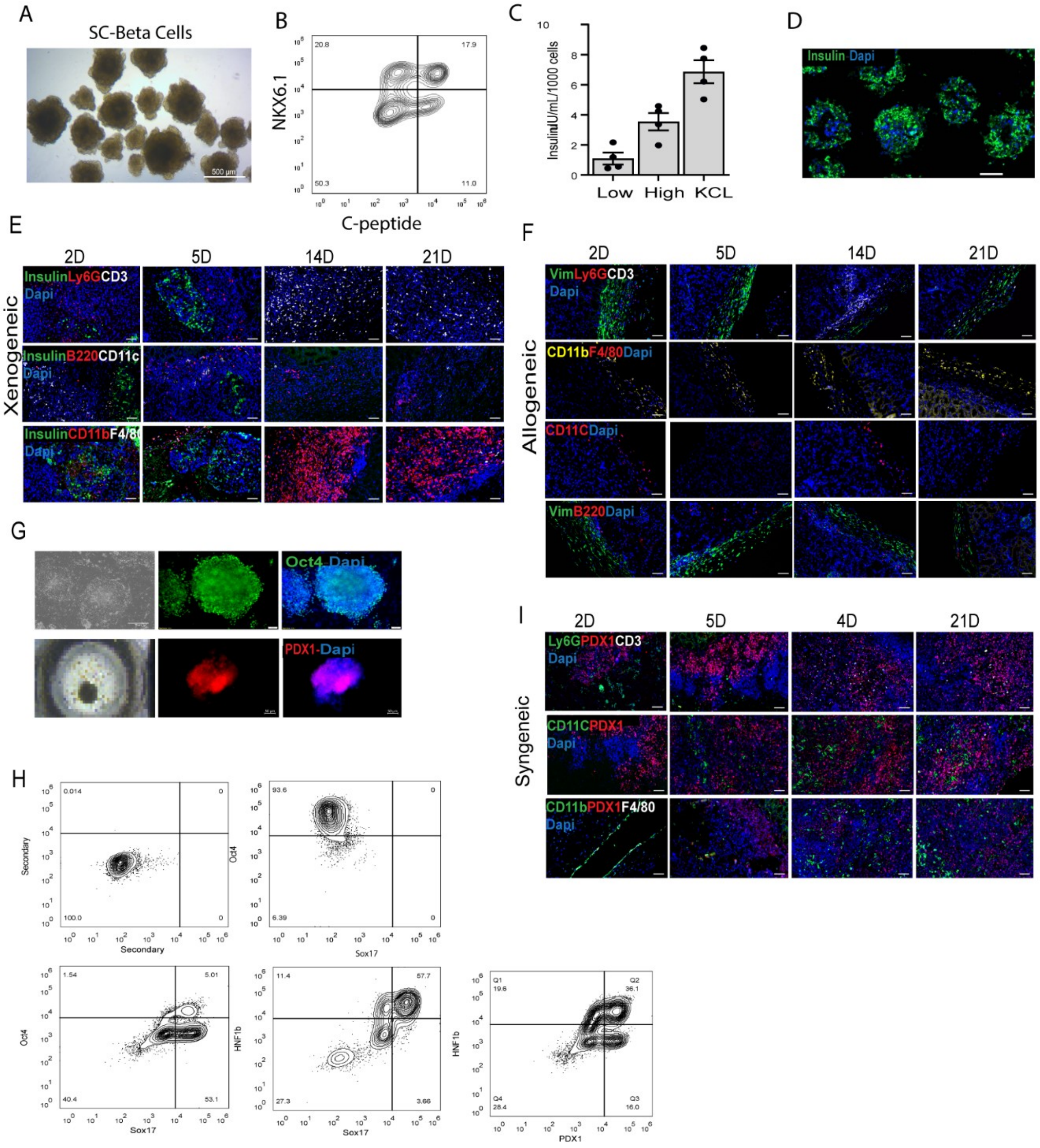

Figure S1. Characterization of SC-β cells and immune cell infiltration in xenogeneic, allogeneic, and syngeneic settings. (A) Brightfield image showing the clustered morphology of SC-β cells. (B) Flow cytometry plot confirming co-expression of C-peptide and NKX6.1 in differentiated SC-β cells. (C) Glucose-stimulated insulin secretion (GSIS) assay showing increased insulin release under high-glucose (16.5 mM) and KCl (30 mM) conditions compared with low-glucose conditions (3.3 mM). Data are presented as mean ± SEM. (D) Immunofluorescence image of SC-β clusters stained for insulin (green), with nuclei counterstained with DAPI (blue). (E, F,I) Immunofluorescence staining of grafts retrieved from xenogeneic, allogeneic, and syngeneic transplantation models at 2, 5, 14, and 21 days after transplantation. In xenogeneic grafts (E), Ly6G, CD11b, and B220 were shown in red, whereas CD11c, CD3, and F4/80 were shown in white. In allogeneic grafts (F), vimentin was shown in green; Ly6G, F4/80,

CD11c, and B220 in red; CD11b in yellow; and nuclei in blue with DAPI. In syngeneic grafts (I), Ly6G, CD11c, and CD11b were shown in green; PDX1 in red; CD3 and F4/80 in white; and nuclei in blue with DAPI. (G) For syngeneic transplantation, mouse iPSCs (miPSCs) were cultured on MEFs, formed spheroids, and stained for OCT4 (green) to confirm pluripotency. Following directed differentiation, pancreatic progenitors were confirmed by PDX1 staining (red). (H) Flow cytometry analysis of differentiated pancreatic progenitors showing the secondary-antibody control, OCT4 expression in pluripotent cells, SOX17 and HNF1β expression at the definitive endoderm stage, and PDX1 expression in pancreatic progenitor cells. Images were acquired at 20× magnification; scale bars represent 50 μm.

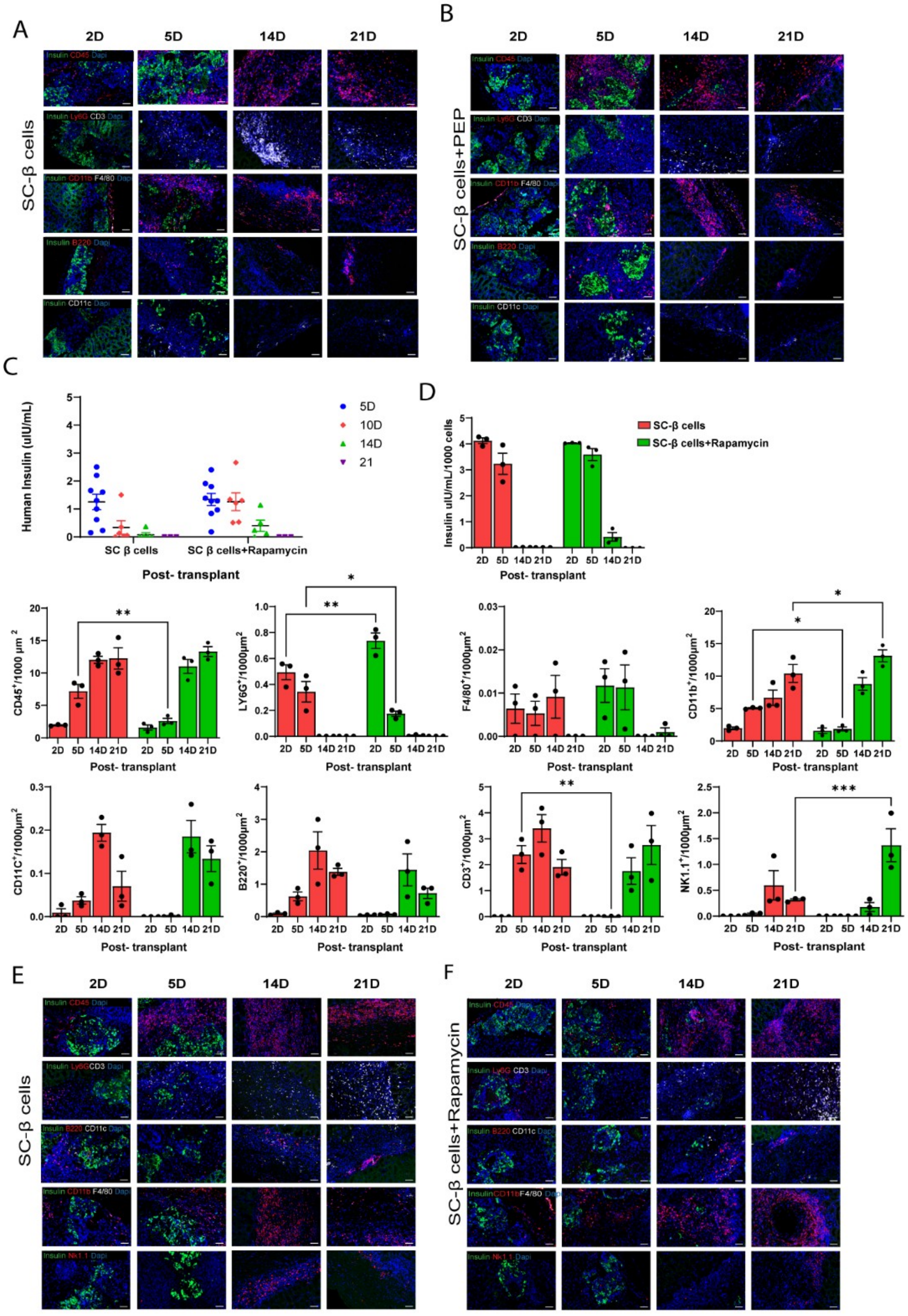

Figure **S2.** Co-transplantation of PEP with SC-β cells and SC-β cells transplantations with and without rapamycin injection**.** (A, B) Representative immunofluorescence images of xenogeneic grafts following co-transplantation of PEP with SC-β cells. Insulin is shown in green; CD45, Ly6G, CD11b, and B220 are shown in red; and CD3, CD11c, and F4/80 are shown in white. (C) Human insulin levels in the plasma of C57BL/6 mice, measured using a human-specific insulin ELISA on days 5, 10, 14, and 21 after transplantation, with or without rapamycin treatment. (D) Quantification of insulin-positive cells and immune-cell markers within the left kidney capsule at the indicated time points following SC-β cell

transplantation with or without rapamycin treatment. (E,F) Representative immunofluorescence images of xenogeneic grafts following SC-β cell transplantation with or without rapamycin treatment. Insulin is shown in green; CD45, Ly6G, CD11b, NK1.1, and B220 are shown in red; and CD3, CD11c, and F4/80 are shown in white. Images were acquired at 20× magnification; scale bars represent 50 μm. Statistical analysis was performed using an unpaired Student's t-test for two-group comparisons and two-way ANOVA followed by multiple-comparison testing for multigroup comparisons. Statistical significance is indicated as *$p < 0.05$, **$p < 0.01$, and ***$p < 0.001$.

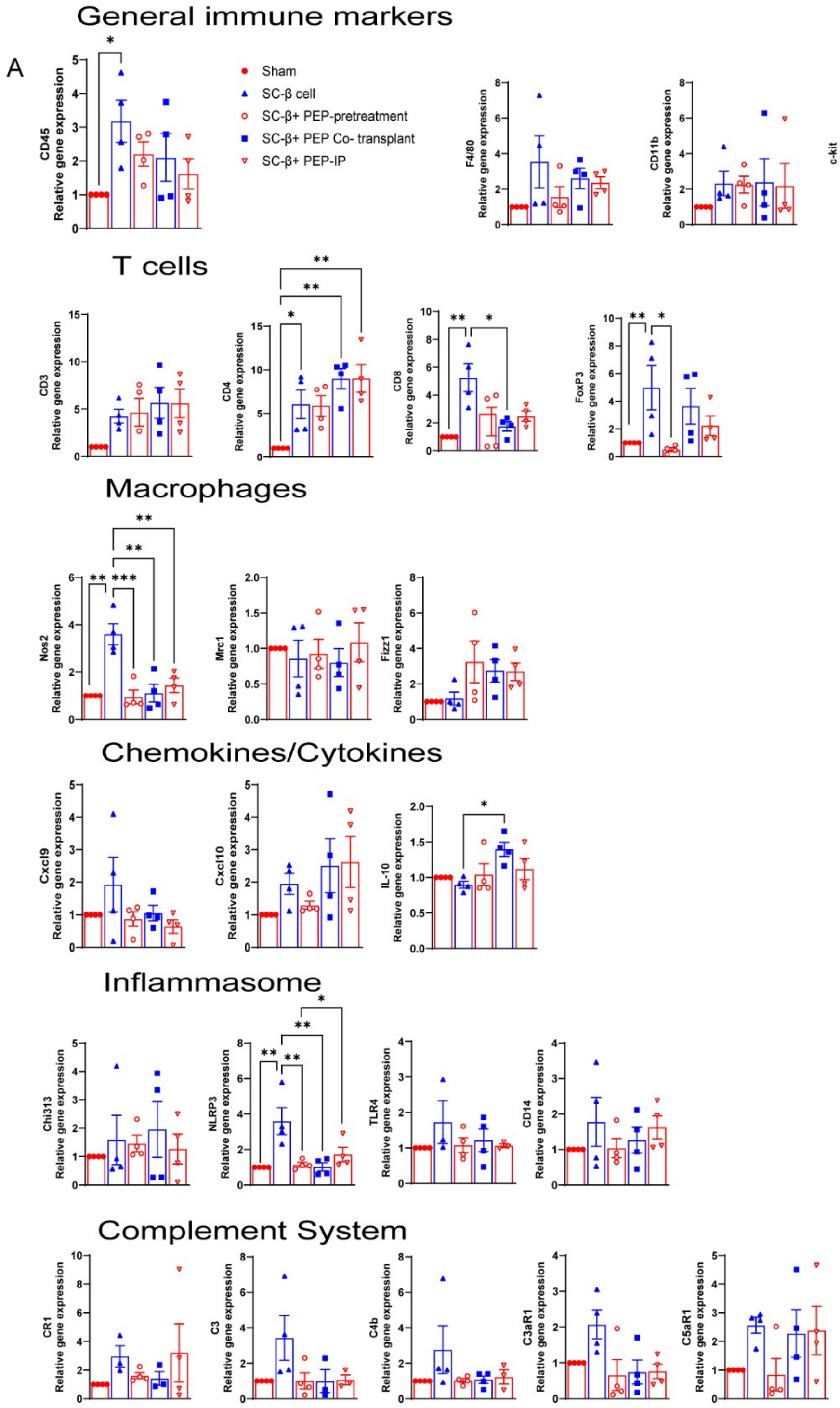

Figure S3. PEP delays xenograft rejection and limits NK cell recruitment across multiple administration routes. (A) Relative gene expression analysis of immune-related markers in graft-bearing kidneys from sham, SC-β cell, SC-β + PEP pretreatment, SC-β + PEP co-transplant, and SC-β + PEP intraperitoneal (IP) groups. Markers are grouped into general immune markers, T cell–associated markers, macrophage-associated markers, chemokines/cytokines, inflammasome-related genes, and complement system-related

genes. Data are presented as relative gene expressions normalized to sham controls and expressed as mean ± SEM. Statistical analysis was performed using one-way ANOVA followed by multiple-comparison testing. Statistical significance is indicated as * $p < 0.05$, ** $p < 0.01$, and ***$p < 0.001$.

Supplementary Table S1. Antibodies used in IF staining

Proteins	Vendor and Cat.no.	Dilution
Insulin	Invitrogen, PA1-26938	1:250
Insulin	Cell signaling, 4590S	1:200
NKX6.1	DSHB, F55A12	1:100
Sox17	R &D AF1924	1:250
Oct4	Sanda Cruz Biotechnology, SC-5279	1:250
HNF1B	Sanda Cruz Biotechnology, SC-130407	1:100
PDX1	R&D Systems, AF2419	1:250
Vimentin	Cell signaling, D21H3	1:250
CD45	Thermo Fisher 14045282	1:500
CD11b	Abcam, ab133357	1:500
F4/80	Bio-Rad, MCA497G	1:500
CD11c	Novus Biologicals, NB110-97871	1:500
Ly6G	Bio legend, 128015	1:250
CD3	Cell signaling Tech, D4V8L	1:100
B220	Invitrogen, RA3-6B2	1:250
NK1.1	Invitrogen, PK136	1:200

Supplementary Table S2. RT-PCR primers

Gene	Catalog #	Amplicon Length (exons)
HPRT	Mm03024075_m1	131
CD45	Mm00448522_m1	60
CD11b	Mm00434455_m1	69
F4/80	Mm00802529_m1	92
cKit	Mm00445212_m1	71
CD3e	Mm01179194	121
CD4	Mm00442754_m1	78
CD8	Mm01182107_g1	68
FoxP3	Mm00475162_m1	74
Cxcl9	Mm00434946_m1	64
Cxcl10	Mm00445235_m1	59
NK1.1	Mm07307455_m1	92
IL-10	Mm01288386_m1	136
Nos2	Mm00440502_m1	66
Mrc1	Mm00485148_m1	76
Fizz1	Mm00445109_m1	86
Chi313	Mm00657889_m1	146
Nlrp3	Mm00840904_m1	84
TLR4	Mm00445273_m1	65
CD14	Mm00438094_g1	69
CR1	Mm00801681_m1	77
C3	Mm01232779_m1	88
C4b	Mm00437893_g1	73
C3aR1	Mm02620006_s1	120
C5aR1	Mm00500292_s1	69

Supplementary Table S3. RT-PCR primers for PBMCs

Genes	5' Sequences	3' Sequences
GAPDH	ATCACCATCTTCCAGGAGCGA	TTCTCCATGGTGGTGAAGACG
CD45	GCAAAGATGCCCAGTGTTCCACTT	ATCTGAGGTGTTCGCTGTGATGGT
CD3	CAGAGGAAGCAAACCAGAAGA	GTGATGCAGATGTCCACTATGA
CD8	GTGCAACAGCCACTACATTAC	ATGAATACACAGGGAGGAAGAC
CD4	CCTTTGGGACCAAGGAAACT	CAATTGCACCTCCTCCTTCT
NK1.1	CTACAGGTTCAAGGCCAAC	CCACTCAGA AAGCACAGTCA
Granzyme B	GCCAGCAGAAGCAGGATTGGT	ACTGAGCCTGCTCCACCTCTTT
EOMES	CCTTCTTCCAGCGTGTGAGC	AAGGAAAGCGCCGTGAGTTG
KLRK1	ACCTCCGACCACGAATCCAC	TCAAGATCTTCCCTCTCTGAGCA
FoxP3	CACGCATGTTTGCCTTCTTC	CTTGTGCAGACTCAGGTTGT
Nos2	AAATCCCACCTGACCTTGTG	GAGAAGGGCTGTGTGTTGAA
FIZZ1	CTTGCCTCCTTCTCATCCTAATC	CCATAACGGAGTCTAAGGAACAC
IL-10	CTTCAGCAGAGTGAAGACTTTCT	GCAACCCAGGTAACCCTTAAA
F4/80	CACATCTGTGCCTTTGGTTAT	TTCAGCCAGCAGCGATTAT
